# Risk analyses for perioperative morbidities after aseptic knee revision arthroplasty

**DOI:** 10.1007/s00402-024-05654-2

**Published:** 2024-12-21

**Authors:** Ahmed Abdelghany, Filippo Migliorini, Christian Peterlein, Christian Götze, Julian Koettnitz

**Affiliations:** 1 Department of General Orthopaedics, Auguste-Viktoria-Clinic Bad Oeynhausen, University Hospital of RUB-Bochum, Am Kokturkanal, 32545 Bad Oeynhausen, Germany; 2 Department of Orthopaedics and Trauma Surgery, University Clinic Aachen, RWTH Aachen University Clinic, 52064 Aachen, Germany; 3 Ruhr-University Bochum, Universitätsstraße 150, 44801 Bochum, Germany

**Keywords:** Age, Aseptic revision TKA, Blood transfusion, C-reactive protein, Perioperative complications, Gender

## Abstract

**Introduction:**

Total lower limb arthroplasties are standard orthopedic surgeries that are steadily increasing in modern civilization. In proportion, the number of revision arthroplasties and the corresponding financial burden for healthcare systems will increase. The present clinical investigation analyzed morbidities after aseptic revision knee arthroplasty.

**Methods:**

Data from 122 patients following aseptic revision TKA (total knee arthroplasty) were collected. The study collected information on systemic and surgical complications during hospitalization and follow-up, blood transfusion units, and changes in Hb and CRP levels. Hb and CRP were measured preoperatively and on postoperative days 1, 2, and 4. Statistical analyses were performed using IBM SPSS version 29.

**Results:**

Significant differences were observed in systemic, surgery-related and blood transfusion rates and reasons for knee replacement changes based on age and gender. Of the patients, 38.5% received a full component change, 11.5% received a partial component replacement (either femoral or tibial), 30.3% had an isolated inlay change, and 19.7% underwent debridement only. Femoral and tibial loosening were more frequent in patients over 75 years and those with longer intervals between the initial operation and re-presentation (p = 0.001). Patients with movement deficits and malposition presented earlier for consultation (p = 0.02). Prolonged high CRP levels were significantly correlated with systemic complications, longer hospital stays, and extended operative times.

**Conclusions:**

Aseptic knee revision arthroplasty is a complex procedure with perioperative morbidities that can significantly impact outcomes. Age and gender are crucial factors in managing complications during hospitalization and follow-up. Elderly patients, particularly those over 75 years, are more prone to aseptic loosening and require tailored preoperative preparation. The study underscores the importance of individualized patient care strategies to mitigate risks and improve outcomes in aseptic knee revision arthroplasty.

**Trial registration:** Not applicable.

## Introduction

Aseptic loosening remains one of the leading causes for revision surgery in total knee arthroplasty (TKA). Despite advances in implant design and surgical techniques, the number of revision TKAs continues to rise globally, posing significant clinical and economic challenges [1]. The pathogenesis of aseptic loosening is multifactorial, involving mechanical, biological, and patient-related factors. Wear particle-induced osteolysis remains a central mechanism, but other contributors include stress shielding, micromotion, and patient factors such as obesity and high activity levels. Early identification of aseptic loosening is crucial for optimal management, yet diagnosis can be challenging, often requiring a combination of clinical, radiographic, and laboratory evaluations [2]. Aseptic revision TKA presents unique challenges compared to primary TKA, including bone loss, ligament instability, and the need for more complex implant systems. The goals of revision surgery are to relieve pain, restore function, and achieve long-term implant survivorship. However, outcomes of revision TKA are generally inferior to those of primary TKA, with higher complication and re-revision rates [3, 4]. Recent advances in implant design, surgical techniques, and perioperative management have aimed to improve the results of aseptic revision TKA. These include modular revision implant systems, augments to address bone defects, and more constrained prosthesis options for cases with ligamentous insufficiency. Additionally, there is a controversial debate regarding the optimal extent of component revision, whether to revise all components or perform isolated component revision for selected patients [5, 6]. Furthermore, a close view on the costs of revision knee arthroplasty reveals an enormous burden on the healthcare system. In some regions, annual costs of about one million pounds are described [7]. The primary objective of this study is to investigate and analyze the systemic and surgical complications associated with aseptic revision total knee arthroplasty (TKA). Aseptic revision TKA is necessitated by mechanical issues such as implant loosening, instability, or fractures, which occur without the presence of infection. This study aims to identify the frequency and types of complications and to determine the factors that increase the risk of these complications. As gender analyses in this field are uncommon, a precise analyses of gender significant differences in combination with age analyses have been done.

## Methods

### Study design

The present study was performed according to Strengthening the Reporting of Observational Studies in Epidemiology (STROBE) [25]. This study was conducted at the Department of Orthopedic Surgery of a University Hospital in Germany. The study was conducted in accordance with the Declaration of Helsinki and approved by the local Ethics Committee of the university hospital.

Data from patients who underwent aseptic revision TKA during 2018 to 2020 were retrieved. The data were retrieved using Pegasos 7 (Nexus Marabu GmbH, Berlin, Germany) and collected in Microsoft Excel (Microsoft Corporation, Redmond, WA, USA). The following data were collected at admission: age, sex, side, body mass index (BMI), length of hospital stay, length of intensive care unit stay, and American Society of Anesthesiologists physical status (ASA). The ASA classification counts from 1–6 (normal health, mild, severe, severe with life-threatening conditions, moribund diseases, and brain dead) [26]. The following data were collected during the hospitalization: complete knee replacement or partial component replacement (femoral or tibial replacement), preoperative and postoperative hemoglobin (Hb), the incidence of systemic and surgical complications, and the frequency of blood unit transfusions. Systemic complications included pulmonary, cardiac, urogenital, and neurologic complications. Surgical-related complications included early infections, neurologic disorders (paresthesia and periphery paralyses), fractures, bleeding, aseptic loosening, surgical interventions post-surgery, and post-discharge complications such as infections or instabilities. If patient data was not accessible, the patient was excluded from the present investigation.

### Recruitment process

In total, data from 122 patients with aseptic revision TKA were retrieved from 2018 to 2020.

### Eligibility criteria

All patients undergoing aseptic revision TKA were retrieved, and their eligibility was assessed. The inclusion criteria were: (1) Patients with primary cemented TKA; (2) patients aged between 40 and 100 years; (3) accessible patient data; and (4) revision surgery. The exclusion criteria were: (1) septic revision surgery; (2) any blood abnormalities; and (3) a peripheral arteriovenous or neurologic ailment.

### Perioperative management

The revision arthroplasties were conducted with Smith & Nephew Genesis II PS system, when changing from CR to PS was possible and Legion Knee Revision system, when intramedullary stability was necessary and Link Endo-Model Knee System, when additionally collateral stability was needed. No tranexamic acid was given. For revision TKA the medial parapatellar approach was used. All patients received general anesthesia and preoperative femoral nerve blocks, despite of any allergies against the medication. A stationary or ambulant rehabilitation program was organized prior to hospital release.

### Blood unit supply

The indication for the blood unit transfusion was according to the restrictive Cochrane guidelines: Hb-levels over 8.0 g/dL indicated no transfusion; between 7 and 9 with concomitant clinical symptoms such as dizziness, nausea, malaise, or loss of appetite, and Hb-levels under 8 g/dL indicated transfusion [27].

### Statistical analyses

All statistical analyses were performed using the software IBM SPSS version 29 (IBM, Armonk, NY, USA). Metric-scaled data were analyzed by mean, standard deviation, and variance. Nominal, dichotomous data were analyzed by Fisher’s exact test. For the analysis of metric and nominally scaled variables, the T-test for independent samples, variance analyses, the Levene test, and the Welch tests were used. Cohen’s d (small 0.20; medium 0.50; large 0.80) and 95% interval were used as effect sizes. The effect size used was phi (small 0.10; medium 0.30; large 0.50). For correlation analyses of metrical and ordinal scales the bivariat correlation pearson and spearman analyses were used. For nominal and ordinal scales, the Mann–whitney-U-Test and Kruskal–Wallis-Test was used. The significance level was set to two-sided with α = 0.05.

## Results

### Patient demographics

Gender (p = 0.434), time of operation (p = 0.839), number of previous diseases (p = 0.403), and ASA score (p = 0.085) were not significantly different, and the BMI (p = 0.001) was significantly different between < 75 years old and > 75 years old patients (Table 1).

**Table 1:** Patient demographics

Demographics	
Age	68.1 ± 9.7 years
Men	41.8% (51 of 122)
Women	58.2% (71 of 122)
cCRA	38.5% (47 of 122)
pCRA	11.0% (14 of 122)
BMI	31.17 ± 5.7 kg/m^2^
ASA score	1.9 ± 0.67
Pre-diseases	2.6 ± 1.9
Surgery time	121 ± 56.91 min
The length of hospital stay (d)	12.7 ± 3.9
≥ 75 years BMI	28.5 ± 4.1
≥ 75 years length of hospital stay (d)	14.5 ± 4.4
≥ 75 years surgery time	115 ± 38.1 min
≥ 75 years Pre-diseases	3.0 ± 2.5

*cCRA* complete component revision arthroplasty, *pCRA* partial component revision ar-throplasty, *d* = days

Age (p = 0.114), length of hospital stay (p = 0.574), time of operation (p = 0.824) and the number of Pre-diseases (p = 0.114) were not significantly different, and the BMI (p = 0.002) was significantly different for female and male gender. For component changes see Table 2.

**Table 2:** Component changes in relation to age and gender

	pCRA	cCRA	Inlay only	Other
Gender				
Male (n = 51)	6 (11.7%)	18 (35.2%)	13 (25.4%)	14 (27.4%)
Female (n = 71)	8 (11.2%)	29 (40.8%)	24 (33.0%)	10 (14.0%)
Age				
Age < 75 yrs (n = 86)	9 (10.4%)	29 (33.7%)	34 (39.5%)	14 (16.2%)
Age > 75 yrs (n = 36)	5 (13.8%)	18 (50%)	3 (8.3%)	10 (27.7%)

‘Other’ includes debridement only, Axis-bushing replacement, patella replacement

*cCRA* complete component revision arthroplasty, *pCRA* partial component revision arthroplasty, *n* 122 patients, *yrs* years

Patients > 75 years old received significantly more often a tibial change (p = 0.047) whereas femoral changes were not significantly different (p = 0.098). For the reasons of replacement of the arthroplasty, elderly patients above 75 years were more often related to femoral and tibial loosening (p = 0.041; p = 0.001). Other reasons of replacement were not significantly different between the age groups (Table 3).

**Table 3:** Perioperative surgery-related complications after aseptic revision surgery

Surgery-Related Complications	Age groups (p)		Gender (p)
Intraoperative injuries			
Tibial tuberosity fracture	0.122		0.418
Rupture of quadriceps tendon	1.0		1.0
Hematoma		0.028*	1.0
Surgery-associated infections			
Wound healing disorder (> 7 d)	0.043*		0.418
Others			
Periarticular ossifications	0.518		0.418
surgical interventions during hospitalisation**	0.028*		0.173

*Significant difference; **number of interventions after revision arthroplasty (re-revision with or without component changes)

### Perioperative complications

No nerve or vascular injuries, intraoperative femur, tibial shaft, patella fractures and periprosthetic joint infections were documented in the 122 patients. Significant differences were found for the occurrence of surgical complications and the age groups (p = 0.012; r = 0.228), but not for the gender groups. No significant differences could be found in documented late surgical complications (at least 6 months after revision surgery) for the age and gender groups. In total, in 17 patients (13.95%) late surgical complications occurred (see Table 4 for more details).

**Table 4:** Number of patients and type of late surgical complications (more than 6 months after revision surgery and hospital stay)

PPF	Instability	Quadriceps tendon rupture	hematoma	Restriction of movement	thrombosis	Patella luxation	PPI
3 2.4%	1 0.8%	1 0.8%	1 0.8%	1 0.8%	1 0.8%	4 0.8%	5 0.8%

*PPF* periprosthetic fracture, *PPI* periprosthetic infection

For systemic complications no significant difference was found between the frequency of systemic complications and age or gender groups (p = 0.403; p = 0.598). The investigation of previous illnesses and age in relation to the occurrence of systemic complications revealed significant correlations. The more pre-existing conditions in patients, the more frequently systemic complications occurred (p = 0.001, r = 0.291). Pre-existing conditions alone had no statistical association with the occurrence of systemic complications. No Embolism, pneumonia, myocardial infarction or Apoplex occurred. For more details see Table 5.

**Table 5:** Perioperative systemic complications after aseptic revision surgery

Systemic complications	Age groups (p)	Gender (p)
Urogenital		
Urinary tract infection	0.363	0.636
Cardiac/Vascular		
Thrombosis	0.122	1.0
Arrhythmia	0.518	0.518
Pulmonary		
Pulmonary edema	0.122	0.395
Neurological		
Transitory psychotic syndrome	1.0	0.235

The laboratory chemical signs of inflammation in the form of C reactive protein at the start of therapy were on average 7.43 mg/l and at the day of discharge (about 7 days after surgery) 52.83 mg/l. At day 1, 2 and 4 after operation the mean values were 24.78 mg/l, 83.69 mg/l and 92.92 mg/l. Prolonged high CRP-levels correlated significantly with systemic complications, the length of hospital stay, and time of operation. More details see Table 6.

**Table 6:** Correlation of CRP levels and complications, postoperative transfusions, time of operations, length of stay and age

		CRP levels ≥ 7 days after surgery
Surgery-related complications	Pearson-Correlation	0.031
	Significance (2-sided)	0.750
	N	106
Systemic complications	Pearson-Correlation	0.272**
	Significance (2-sided)	0.005
	N	106
Postoperative Transfusions	Pearson-Correlation	0.367**
	Significance (2-sided)	0.001
	N	105
Time of operation	Pearson-Correlation	0.265**
	Significance (2-sided)	0.006
	N	106
Length of hospital stay	Pearson-Correlation	0.369**
	Significance (2-sided)	0.001
	N	106
Patients age	Pearson-Correlation	0.142**
	Significance (2-sided)	0.007
	N	122

**The correlation is significant at the level 0.01 (2-sided)

### Hb drop and blood transfusion

Hb was collected preoperatively, postoperatively (in the evening) and on POD (postoperative day) 1, 2, and 4 and 7 after surgery. The mean preoperative hemoglobin value of all patients was 135.27 mg/dl, the mean last postoperative hemoglobin value was 100.94 mg/dl.

**Fig. 1:**
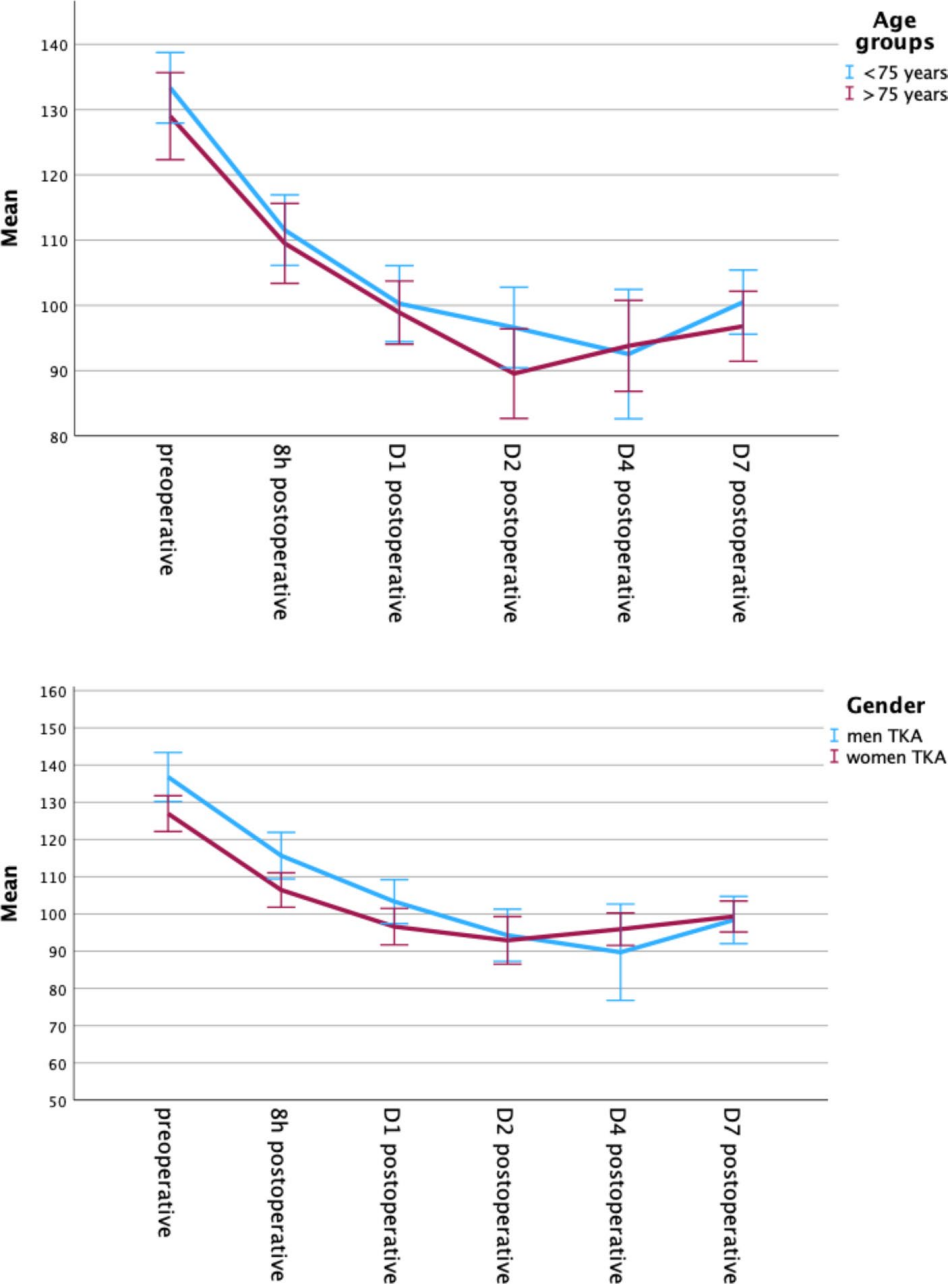
Hemoglobin progression age groups. Hemoglobin progression gender groups

There was no significant difference in hemoglobin progression between the age or gender groups (p = 0.371; p = 0.296). Figure 1 Hb drops under 80 mg/dl were found in 19 patients (15.6%), of which 63.3% were women and 36.7% were men (p = 0.686). 25% of the patients > 75 years of age experienced a Hb drop under 80 mg/dl. Of course, patients with hemoglobin under 80 mg/dl received significantly more blood units than the other patients (p = 0.001). Patients > 75 years received significantly more often blood units (Cohen’s d = 0.533, p = 0.026) than the younger group. No significant gender differences were found. Age and gender were significant factors influencing the perioperative outcomes.

## Discussion

The perioperative morbidities associated with aseptic knee revision arthroplasty (TKA) present a complex interplay of systemic and surgery-related complications, influenced by patient demographics, comorbidities, and procedural specifics. This discussion synthesizes findings from the current study and relevant literature to provide a comprehensive understanding of these risks. This study identified significant perioperative complications in patients undergoing aseptic knee revision arthroplasty. The most common complications included systemic issues such as pulmonary, cardiac, and urogenital complications, as well as surgery-related complications like hematomas, wound healing disorders, and the need for additional surgical interventions. These findings are consistent with previous studies that highlight the high incidence of perioperative morbidity in TKA procedures [8, 9].

Age and gender were significant factors influencing the perioperative outcomes. Older patients, particularly those over 75 years, exhibited higher rates of complications, including femoral and tibial loosening, which necessitated more frequent tibial component changes. This is in line with previous research indicating that age is a critical risk factor for complications in TKA [10, 11]. It should be noted that older patients may have had a primary prosthesis for longer than younger patients who have had a revision. Gender differences were also noted, with women experiencing higher rates of wound healing disorders and neurologic complications postoperatively. This aligns with findings from other studies that suggest women are at greater risk for certain postoperative complications following arthroplasties [12–16]. Hemoglobin levels and the need for blood transfusions were important perioperative considerations. This study found no significant differences in hemoglobin progression between age or gender groups, but older patients required more blood transfusions. This is confirmed by Koch et al. (2023), indicating that perioperative blood management is crucial in TKA, with restrictive transfusion protocols being recommended to minimize complications [17]. CRP levels were monitored as a marker for systemic inflammation and potential complications. Prolonged high CRP levels were significantly correlated with systemic complications, longer hospital stays, and extended operative times. This finding is supported by other studies that have identified elevated CRP as a predictor of postoperative complications [18]. For example, Shen et al. (2017) evaluated serum CRP levels after TKA and found that CRP levels are closely linked to the extent of surgical trauma. The study highlighted that CRP is a sensitive marker for inflammation and infection, with levels peaking within the first 48 h postoperatively and gradually declining thereafter [19]. This study has several limitations, including its retrospective design and the potential for missing data due to incomplete hospital records. Additionally, the study did not set an age limit, which could introduce bias. Future research should focus on prospective studies with larger sample sizes to validate these findings and explore the development of predictive models that incorporate multiple risk factors, including patient demographics, comorbidities, and perioperative markers like CRP and hemoglobin levels.

## Conclusions

In conclusion, perioperative morbidities after aseptic knee revision arthroplasty are influenced by a range of factors including age, gender, and systemic health markers. Effective perioperative management and individualized patient care strategies are essential to mitigate these risks. Further research is needed to refine predictive models and improve outcomes for patients undergoing this complex procedure.

## Data Availability

The datasets used and analyzed during the current study are available from the corresponding author on reasonable request.

## Abbreviations

ASA: The American society of anesthesiology
BMI: Body mass index
Hb: Hemoglobin
USD: US-Dollar
TKA: Total knee arthroplasty
